# Associations between Parental Anxiety/Depression and Child Behavior Problems Related to Autism Spectrum Disorders: The Roles of Parenting Stress and Parenting Self-Efficacy

**DOI:** 10.1155/2011/395190

**Published:** 2011-12-13

**Authors:** Debra L. Rezendes, Angela Scarpa

**Affiliations:** Department of Psychology, Virginia Polytechnic Institute and State University, Blacksburg, VA 24061-0002, USA

## Abstract

Parents of children with autism spectrum disorders (ASDs) have been shown to experience increases in stress, depression, and anxiety, which are also associated with child behavior problems related to ASDs. Literature-examining potential mechanisms that underlie the relationship of child behavior problems and parental anxiety/depression in this population are scarce. The current study sought to examine the roles of parenting stress and parenting self-efficacy as mediators between child behavior problems and parental anxiety/depression. Using a sample of 134 mothers, these potential mediators were tested. Hypotheses were supported, indicating that parenting stress mediated the relationship between child behavior problems and decreased parenting self-efficacy, and decreased parenting self-efficacy in turn partially mediated the relationship between parenting stress and increased depression/anxiety.

## 1. Introduction

Autism spectrum disorders (ASDs) are lifelong neurodevelopmental disabilities that begin in infancy and impair communication and social behaviors. Recent estimates of the prevalence of autism suggest that the disorder is increasing, with as many as 1 in 110 children diagnosed with an ASD in the United States [1]. With the incidence of ASD on the rise, it becomes important to understand how these disorders affect the parent-child relationship, including reciprocal associations between parent emotions and child behavior.

Several factors have been shown to work in concert to increase stress in parents of children with ASD. First and foremost, the realization that there is no cure for the disorder may serve to increase parenting stress [2]. Aspects of the child's behavior, specifically socially inappropriate and aggressive behaviors typically associated with ASD, have been found to be associated with increases in parenting stress [3, 4], as well as being confronted by antipathy for their child's behaviors [5] due to a lack of understanding of ASD [6]. Additionally, raising a child with ASD typically involves allocating extra time to meet the needs of the child [7]. These findings suggest that multiple changes occur in the parental role to accommodate the challenges of raising a child with ASD. While examining such changes is helpful in increasing our understanding of parenting stressors, examining the interplay of both parental and child factors as they contribute to the parent-child relationship (in terms of parental emotions and child behavior problems) will provide a greater understanding of the types of support and potential interventions needed by families of children with ASD. 

### 1.1. Autism Spectrum Disorders and Parental Depression/Anxiety

Much of the prior research has focused on parenting stressors related to raising a child with ASD and has indicated a relationship between parental anxiety/depression and the behavior of children with a pervasive developmental disorder, including autistic disorder, Asperger's disorder, and pervasive developmental disorder-not otherwise specified (PDD-NOS). In particular, this research suggests that mothers of children with autism report more depressive symptoms than fathers [8], who in turn have more depressive symptoms than controls [9]. In fact, the lifetime prevalence of major depressive disorder may be higher in the parents of children with autism than parents of children with Down syndrome [10]. Mothers of youth with ASD also report experiencing excessive anxiety [11]. In another study, nearly half of the parents of youth with ASD were found to be severely anxious, and nearly two-thirds were found to be clinically depressed [12]. 

The amount of stress in parents of children with developmental disabilities appears strongly related to increases in their depressive symptoms and to decreases in their psychological well-being [13–16]. Child behavioral problems in particular, and not the severity of the disorder, have been found to contribute to maternal depressive symptoms; additionally, a child's lack of prosocial behaviors contributes to maternal stress [17]. Other studies indicate that behavior problems in youth with ASD also predict the level of maternal anxiety and stress experienced [18, 19], as well as maternal mood disorders and depression [20–23]. These findings suggest that the child's behavioral problems produce negative effects on the parents' psychological well-being. While the relationship between child behavior problems and negative parental psychological well-being has been established within the current literature, studies examining the mechanisms underlying this relationship are scarce. 

These studies suggest that increases in stressors related to child behavior problems (e.g., harm to oneself or others, destruction of property, and destructive behaviors) can produce an environment more conducive to parental anxiety/depression. In support of a bidirectional relationship, Baker et al. [24] found that parental stressors and child behavior problems work in concert to instigate each other. They found support for a transactional model in which behavior problems resulted in high parenting stress, and high parenting stress resulted in increased behavior problems.

### 1.2. Parenting Self-Efficacy

Prior research suggests that parental factors may influence the relationship between child behavior problems and parenting stress and depression, including the role of parenting self-efficacy within the parenting experience. Pakenham et al. [25] examined the role of self-efficacy within the parental coping process of adjusting to their child's ASD diagnosis and found that parents with better adjustment and coping skills had higher self-efficacy. Other studies found that self-efficacy is negatively associated with parental psychopathology [26, 27]. Kuhn and Carter [28] examined the association between maternal self-efficacy and parental cognitions and found that maternal depression, stress, and guilt all accounted for unique variance in self-efficacy. Mothers who reported higher levels of self-efficacy were also more active in promoting the development of their children, suggesting that self-efficacy may also play a role in parenting behaviors. 

Hastings and Brown [29] found that self-efficacy mediated the effects of child behavior problems on the mother's anxiety and depression. Mothers of children with autism report both increases in parenting stress and decreases in parental competency [30, 31]. Furthermore, mothers who reported feeling more guilt about their child's condition were found to have lower maternal self-efficacy [28]. Current research suggests that parenting self-efficacy may underlie the relationship between child behavior problems and maternal anxiety/depression; however, this direct relationship has not been fully examined.

In the current study, parenting stress refers to the perceived stress of the parent in reaction to the demands of parenting their child, and we suggest that the child's difficult behaviors contribute to the parent's negative mood via decreases in parenting self-efficacy. Since mothers of children with ASD experience higher levels of stress compared to mothers of typical children [32], their increases in parenting stress may reduce parenting self-efficacy, which in turn might lead to increases in anxiety/depression.

### 1.3. Study Aims

This study was designed to assess parental symptoms of anxiety and depression as related to behavioral problems in children and adolescents with ASD, and to examine two potential mechanisms that may underlie this relationship—parenting stress and self-efficacy. Current research does not elucidate possible bidirectional relationships, and thus, the current study aims to try to clarify associations between reciprocal parent-child effects.

Therefore, we tested the following hypotheses. First, we hypothesized that maternal stress would mediate the relationship between child behavior problems and maternal self-efficacy; specifically, it was expected that increases in child behavior problems would be related to increased parenting stress, which would statistically account for decreased parenting self-efficacy. Second, we hypothesized that maternal self-efficacy would mediate the relationship between parenting stress and parental anxiety/depression; specifically, it was expected that increases in parenting stress would be related to decreased parenting self-efficacy, which would statistically account for increased maternal anxiety/depression.

## 2. Method

### 2.1. Participants

Participants consisted of 140 mothers of children between the ages of 3 and 16, diagnosed with ASD, specifically autistic disorder, Asperger's disorder, or PDD-NOS. Four mothers were excluded from analyses because of incomplete responses to over half of the questionnaires, and two mothers were excluded because their children were younger than 36 months. Therefore, only 134 mothers were used in the final analyses. For parents of multiple children with ASD, parents were invited to complete the questionnaires for their oldest child with ASD. Cognitive/intellectual impairment was not an exclusion criteria for the current study, in order to allow for the full spectrum of ASD to be included. There were no other inclusion or exclusion criteria.

The average age of the mothers completing the study was 39.01 years (SD = 8.01). A majority of the parents were self-reported as Caucasian (94.2%), followed by African-American (2.2%), Latino (1.4%), native American (1.4%), and Asian/Island pacifier (.7%). Mothers most often reported having some college education (29.5%), followed by having a graduate degree (27.5%), having a college degree (25.4%), having a high school degree (10.9%), and completing some graduate studies (6.5%); one participant did not answer this question. 

The average age of children with ASD was approximately 9 years, 2 months, with ages ranging from 3 years to 16 years, and 10 months. A majority of the children were boys (79.9%). Most of the children were reported as being diagnosed with autism (56.9%), followed by PDD-NOS (22.0), and Asperger's disorder (21.2%). Approximately, half of the children (48.9%) had been diagnosed with a comorbid disorder (e.g., attention-deficit hyperactivity disorder, anxiety disorders, oppositional defiant disorder, bipolar Disorder, cognitive impairments, speech impairments and sensory disorders). On average, children were 4 years, 2 months when diagnosed with ASD, and it had been on average 4 years, 8 months since their diagnosis. 16.3% of the children also had a sibling diagnosed with ASD. Most children were reportedly diagnosed by a physician (63.7%), followed by a psychologist (25.4%), a team of specialists or hospital/clinic (7.2%), or a school (2%), and the remainder did not indicate who diagnosed their child. For detailed parent/child characteristics, refer to Table 1. 

The participants were recruited via flyers/announcements sent to local and national autism groups and organizations, and they were directed to a website where they could complete the following measures online.

### 2.2. Measures

#### 2.2.1. Demographics Questionnaire

The demographic questionnaire contained questions regarding parent and child information (i.e., race, age, education status, and income level), as well as information about the child's diagnosis (i.e., age at which child was diagnosed, child's diagnosis, type of professional who made the diagnosis, and current autism symptoms exhibited by the child). Regarding severity of ASD, parents were asked to select from a list of 56 common symptoms associated with ASD. For the purposes of creating a composite score, reported symptoms were classified into severity ratings for communication (i.e., no verbal language, absent or limited gestures, etc.), reciprocal social interactions (i.e., has trouble joining group, poor eye contact, etc.), and repetitive and restrictive behaviors (i.e., hand flapping, fixation on objects or topics) based on the scoring algorithms of the autism diagnostic observation schedule-generic [33] and the autism diagnostic interview-revised [34]. Items that did not clearly relate to one of these domains were not included in the severity score (i.e., food allergies, gross/fine motor delays, audio/visual learning style, under/oversensitivity to pain, aggressive behaviors, losing/gaining weight, thrush, sleeping habits, anxiety, depression, etc.). The number of items reported by the parent was then summed in each category, creating an overall severity rating for that particular domain (i.e., communication severity, social severity, and repetitive and restricted behaviors severity). The possible range of scores for the domains was as follows: communication severity, 0–6, social severity, 0–6, and reciprocal and restricted behaviors severity, 0–8. Across the study, the average reporting of communication severity was 2.17 (SD = 1.47), social severity was 3.17 (SD = 1.48), and repetitive and restricted behaviors was 2.56 (SD = 1.58). Severity ratings in each domain were then summed to yield a composite severity score, with a possible range of 0–20. The average reporting of overall child severity was 7.89 (SD = 3.56) with scores ranging from 1 to 16. The range of scores is reflective of the heterogeneity of ASD diagnoses within our sample. The Cronbach's alpha for the overall child severity rating was 0.533. Low internal consistency may be accounted for by the heterogeneity of ASD symptoms. 

We also examined the relationship of the severity scores with the child's diagnosis to ensure that the severity score reflected child functioning levels typically seen in children with autism, Asperger's disorder, and PDD-NOS. There was a significant difference in the overall severity scores across diagnostic groups, *F* (2, 133) = 9.56, *P* = .001, whereby post hoc Tukey HSD tests indicated children diagnosed with autism had significantly higher severity scores (*M* = 8.94) than children diagnosed with Asperger's disorder (*M* = 6.97) and children diagnosed with PDD-NOS (*M* = 6.40). There was no significant difference in severity scores between children diagnosed with Asperger's disorder and children diagnosed with PDD-NOS.

#### 2.2.2. Depression Anxiety Stress Scale (DASS)

A 42-item self-report questionnaire that measures depression, anxiety, and tension/stress on a scale from 0 *did not apply to me at all, *to 3, *applied to me very much or most of the time*. The depression/anxiety scales account for 28 of the total items. Reliability for the three scales, depression (0.71), anxiety (0.86), and stress (0.88), is considered good. The depression scale correlates with the beck depression inventory (0.74) and the anxiety scale correlates with the beck anxiety inventory (0.81, [35]). Items for each subscale are totaled to yield a composite score with higher scores indicating more anxiety and depression. For the purposes of the current study, scores were collapsed over the depression and anxiety subscales to yield a total score of parent depression/anxiety. Tension/stress items were excluded to avoid overlap with the parenting stress construct. Across the study, the average reporting of parental depression/anxiety was 14.15 (SD = 13.13) and ranged from 0 to 63.00. The depression anxiety subscale had a Cronbach's alpha of 0.94.

#### 2.2.3. Strengths and Difficulties Questionnaire (SDQ)

A behavioral screening questionnaire, for children of ages 3–16, in which the parent rates the child on 25 attributes that are both positive and negative. Parents are asked to indicate if statements about their child are *not true* (1),* somewhat true* (2),* or certainly true* (3). Items are divided into five categories, each consisting of five statements: conduct problems, hyperactivity, emotional symptoms, peer problems, and prosocial behavior. The questionnaire yields a total score that represents the child's difficulties. Reliability has been shown to be satisfactory on internal consistency (Cronbach's alpha,  0.73), retest stability after 4–6 months (0.62), and cross-informant correlation (0.34; [36]). SDQ scores above the 90th percentile predicted an increase in the probability of independently diagnosed psychiatric disorders (mean odds ratio: 15.7 for parent scales, 15.2 for teacher scales, 6.2 for youth scales; [36]). All subscales, except for the prosocial behavior subscale, are summed to create a total score; higher scores indicate more difficulties. The total difficulties score was used to operationalize child behavior problems. Across the study, the average reporting of child behavioral problems was 19.02 (SD = 5.41). Scores ranged from 7.00 to 36.00. The measure had a Cronbach's Alpha of  .72.

#### 2.2.4. Parenting Sense of Competence Scale (PSOC)

The PSOC was used to assess parenting self-efficacy. The PSOC is a 16-item self-report measure that assesses perceptions of self-competency in the parental role [37, 38]. This questionnaire contains two subscales, parenting self-efficacy and parenting satisfaction, and measures them on a 6-point Likert scale, *strongly disagree *(1) to *strongly agree *(6). For the purposes of the current study, the 8-item self-efficacy subscale was used, which measures the parent's perception of the degree to which she/he has acquired the necessary skills and understanding for the parental role. This subscale has been shown to have an alpha coefficient of  0.80. Scoring for some of the items within this subscale was reversed so that higher scores reflect greater parenting self-efficacy, and then the items are totaled to create an overall score of parenting self-efficacy. Across the study, the average reporting of parenting self-efficacy was 25.64 (SD = 2.86). Scores ranged from 18.00 to 34.00. The parenting self-efficacy subscale had a Cronbach's alpha of  0.69.

#### 2.2.5. Questionnaire on Resources and Stress-Short Form (QRS-SF)

The QRS-SF is a 52-item short-form questionnaire of Holroyd's [39] QRS. It is a parent self-report measure that assesses the family's response to the child's disability [40]. The questionnaire contains four factors: parent and family problems (i.e., problems related to the family functioning or parental well-being that are associated with the child's disability); pessimism (i.e., pessimistic views of the child's future); child characteristics (i.e., the child's impairments and difficulties in various aspects of functioning); physical incapacitation (i.e., physical impairments related to the child's disability). Parents are asked to indicate if a statement is *true* or *false*. Reliability has been shown to be good (Kuder-Richerdson −20 reliability, 0.95). Item-total correlation (0.15–0.63) and interitem correlation (0.26) have also been shown to be satisfactory. Items are summed together to yield a total score with higher scores indicating higher parenting stress. Given that the study's aim was to examine the multidimensional and disability-related stress of parents of children with ASD, the total score was used for analyses. However, to ensure that minimal construct overlap was present, we assessed the relationship of the four QRS subdomains to the SDQ and the DASS. Results are indicated below. The average reporting of parenting stress was 29.32 (SD = 5.18). Scores ranged from 17.00 to 43.00. This measure's Cronbach's alpha was  0.62 (Table 2). 

### 2.3. Procedures

Participants were administered the questionnaires via an online survey. Completion of the survey signified their consent. The total session was estimated to take about 45 minutes.

## 3. Results

### 3.1. Assessing Construct Overlap

Since the QRS total score was used, there was potential that the scale could partly overlap with the behavioral and emotional constructs measured by the SDQ and DASS. Therefore, we assessed the relationship between the four QRS subdomains and the SDQ and DASS. There was no significant relationship between any of the QRS subdomains and the SDQ. Results were as follows: Parent/Family domain, *r*  (133) = .06, *P* = .50; pessimism domain, *r*  (133) = .01, *P* = .88; child characteristics domain, *r*  (133) = .07, *P* = .45; Physical Incapacitation, *r*  (133) = .07, *P* = .45. There was also no significant relationship between the physical incapacitation subdomain of the QRS and the DASS, *r*  (133) = .003, *P* = .97. There were significant relationships between the DASS and the remaining three QRS subdomains (i.e., parent/family, pessimism, and child characteristics). Results were as follows: Parent/Family domain, *r*  (133) = .45, *P* = .001; pessimism domain, *r*  (133) = .39, *P* = .001; child characteristics domain, *r*  (133) = .23, *P* = .007. The significant relationship between the parent/family subdomain and the DASS was expected as prior research has shown a relationship between perceived stress, as measured by the parent/family subdomain of the QRS, and depressive symptoms in parents of children with ASD [18]. The significant relationship between child characteristics and the DASS was also not surprising as prior research has shown that children with autism possess various characteristics that are associated with maternal depression [9]. However, because the child characteristics subdomain is heavily influenced by the current functioning level of the child and therefore may overlap with diagnostic symptoms, the child's diagnosis was used as a covariate in all analyses involving the QRS.

### 3.2. Evaluating Regression Assumptions

The distribution for parental anxiety/depression was negatively skewed. The variable was therefore transformed with a square root transformation and more closely approximated normality, though the distribution was not completely normalized. The transformed variable was used in all following analyses.

### 3.3. Demographic Effects

The associations between demographic factors and the dependent variables (i.e., depression/anxiety, parenting self-efficacy, and child behavior problems) were assessed. Associations between dichotomous variables (i.e., the child's gender, the parent's education, the child's current diagnosis, and time since diagnosis) and the dependent variables were assessed using ANOVA, and associations between continuous variables (i.e., child's and parent's age) and the dependent variables were assessed using Pearson's correlations. 

A significant association between parenting stress and the child's current ASD diagnosis was found, *F*(2,133) = 3.571, *P* = 0.031. Follow-up analyses revealed that parents of children with autism (*M* = 30.19) reported significantly more stress than parents of children with PDD-NOS (*M* = 27.68). A small positive association was also found between parenting stress and maternal age, *r*  (133) = 0.171, *P* = 0.044, such that older mothers reported increased parenting stress. No other significant relationships were found. Because maternal age and the child's current ASD diagnosis were found to have a significant relationship with parenting stress, these variables were retained as covariates within the analyses described later.

### 3.4. Primary Regression Analyses

Using the steps of mediation outlined by Baron and Kenny [41] and Holmbeck [42], a series of regression analyses were conducted to assess parenting stress and parenting self-efficacy as potential mediators. To assess each potential mediator, four regression analyses were conducted. Prior to conducting the analyses, correlations among variables were examined to assess trends in the relationships among variables (see Table 1). Parenting stress was positively associated with depression/anxiety, *r*(133) = 0.50, *P* = 0.001 and child behavior problems *r*  (133) = 0.27, *P* = 0.002, as well as negatively correlated with parenting self-efficacy, *r*  (133) = − 0.168, *P* = 0.049. Additionally, control variables were entered to account for variance by maternal age and ASD diagnosis in regression models examining parenting stress. The first regression analysis examined the relationship between the predictor and the criterion; the second regression analysis examined the relationship between the predictor and the potential mediator; the third regression analysis examined the relationship between the potential mediator and the criterion; the fourth regression analysis examined the effect of the predictor and the potential mediator on the criterion (these analyses are further described below). Based on the hypotheses given, regressions were conducted using one-tailed tests. Power for each regression analysis was calculated using G*Power [43]. Power greater than 80% is typically needed to detect an effect, given the effect exists. Additionally, as a way to further explain the mediation, the proportion of the total effect that was mediated was calculated by multiplying the unstandardized regression coefficients of paths a and b and dividing by the unstandardized regression coefficient of path c [44]. 

First, the potential mediating role of parenting stress between child behavior problems and parenting self-efficacy was tested; maternal age and ASD diagnosis were first entered into the regression equation to control for the effects of these variables on parenting stress. The first equation analysis tested the effect of child behavior problems on parenting self-efficacy, which yielded a significant negative association, *t*  (133) = − 1.83, *P* = 0.035. The second equation tested the effect of child behavioral problems on the potential mediator of parenting stress, which yielded a significant positive association, *t*  (133) = 2.903, *P* = 0.002. The third equation tested the effect of the potential mediator parenting stress on parenting self-efficacy, which yielded a significant negative association, *t*  (133) = −2.48, *P* = 0.0005. The fourth equation tested the effect of child behavior problems and parenting stress on parenting self-efficacy. Parenting stress was significant, *t*  (133) = −2.114, *P* = 0.018, whereas child behavior problems were no longer significant, *t*  (133) = −1.10, *P* = 0.137, suggesting that parenting stress may be accounting for the initial relationship between child behavior problems and parenting self-efficacy. A Sobel test was then conducted to examine the significance of the indirect effect of parenting stress by examining its total effect on the relationship between child behavior problems and parenting self-efficacy (path c) and its direct effect on parenting self-efficacy (path c'). The Sobel test was significant at the one-tailed level (*t* = 1.71, *P* = 0.04), indicating that parenting stress did mediate the relationship between child behavior problems and parenting self-efficacy. Power was approximately 44%, and the total proportion of variance accounted for by the mediator (i.e., parenting stress) was 32.8%. For reporting of *R* square, *R* square change, and betas of each regression equation, please refer to Table 3.

Second, the potential mediating role of parenting self-efficacy between parenting stress and parental anxiety/depression was tested. For all analyses, control variables (i.e., maternal age and child ASD diagnosis) were entered into the models first. The first regression analysis tested the effect of parenting stress on the criterion of parental depression/anxiety, which yielded a significant positive association, *t*  (133) = 5.56, *P* = 0.0005. The second equation tested the effect of parenting stress on the potential mediator of parenting self-efficacy, which yielded a significant negative association, *t*  (133) = −2.48, *P* = 0.0005. The third equation tested the effect of parenting self-efficacy on parental depression/anxiety, which yielded a significant negative association, *t*  (133) = −3.87, *P* = 0.0005. The fourth regression analysis tested the effect of parenting stress and the potential mediator (i.e., parenting self-efficacy) on parental depression/anxiety. Both parenting stress, *t*  (133) = 4.93, *P* = 0.0005 and parenting self-efficacy, *t*  (133) = −3.26, *P* = 0.0005 yielded significant results, indicating a possible partial mediation. Power was approximately 99%. A Sobel test was then conducted to examine the significance of the indirect effect of parenting self-efficacy by examining its total effect on the relationship between parenting stress and parental anxiety/depression (path c) and its direct effect on depression/anxiety (path c'). The Sobel test was significant at the one-tailed level (*t* = 1.86, *P* = 0.03), indicating that parenting self-efficacy did partially mediate the relationship between parenting stress and parental anxiety/depression. The proportion of variance accounted for by the partial mediator (i.e., parenting self-efficacy) was 14.2%. For reporting of *R* square, *R* square change, and betas of each regression equation, please refer to Table 4.

## 4. Discussion

The purpose of the current study was to examine mechanisms that may underlie the relationship between child behavior problems and parental anxiety/depression. It was predicted that (a) parenting stress would mediate the relationship between child behavior problems and parenting self-efficacy and (b) parenting self-efficacy would mediate the relationship between parenting stress and parental depression/anxiety. Overall, the primary findings supported the two hypotheses, indicating that parenting stress mediated the relationship of child behavior problems and parenting self-efficacy, such that child behavior problems were associated with increased parenting stress that in turn accounted for decreased parenting self-efficacy. In addition, the findings indicated that parenting self-efficacy partially mediated the relationship between parenting stress and parental depression/anxiety, such that the resulting decreases in parenting self-efficacy accounted for increased maternal anxiety/depression scores. The findings remained even after controlling for child's diagnosis, reflecting severity of functioning.

### 4.1. Background Variables That May Influence Parenting Stress on Depression/Anxiety

Prior to analyses, the relationship between demographic variables and dependent variables was explored. Two demographic variables were related to parenting stress and subsequently used as control variables but also may be interesting in their own right. 

The child's current ASD diagnosis was also related to mothers' reports of stress. Specifically, mothers of children with an autism diagnosis reported more stress than mothers of children with pervasive developmental disorder-not otherwise specified (PDD-NOS). Children diagnosed with autism tend to display more severe difficulties with social interactions, communication, and repetitive and restricted behaviors. Indeed, results in the current study indicated increased symptoms severity scores in children diagnoses with autism, relative to Asperger's disorder or PDD-NOS. The severity of these behaviors may increase dependence on the parent, thus increasing parenting burden and stress. 

Lastly, a small positive relationship was also found between the maternal age and level of stress, suggesting that older mothers are reporting higher levels of stress. Older mothers may find it more difficult to deal with the demands of raising a child with ASD. Moreover, societal and parental expectations of child development generally assume that independence increases as a child ages. This typical development of independence is often impeded in ASD, and thus the child's caregiving needs and demands may not decrease over time. In turn, the continued needs and demands may serve to increase parenting stress. 

We attempted to address the possible confounds of age and ASD symptom severity by using maternal age and diagnostic status as covariates. It is important to note that our findings on the roles of stress and self-efficacy in the parent-child relationship occurred above and beyond the effects of maternal age or ASD diagnosis. This finding highlights the particular importance of behavior problems associated with ASD in impacting maternal stress, parenting self-efficacy, and negative mood. Such behaviors may be especially stressful for parents and, when chronic or severe, may leave parents questioning their own competence in the parenting role.

### 4.2. Potential Mechanisms Underlying the Relationship between Child Behavior Problems and Maternal Depression/Anxiety

The primary aim of the current study was to examine the potential mechanisms that underlie the relationship between child behavior problems and parental depression/anxiety. Support was found for the mediating role of parenting stress between child behavior problems and parenting self-efficacy. Specifically, child behavior problems were positively associated with increased parenting stress, which in turn accounted for decreased maternal self-efficacy scores. Since ASD symptom severity was also associated with parenting stress, the child's diagnosis was used as a covariate in an attempt to control any construct overlap and/or artificial effects. Even with the introduction of the child's diagnosis as a covariate, the relationships remained significant, providing further support for the mediating role of parenting stress between child behavior problems and parenting self-efficacy. 

The current study found support for the influence of child behavior problems on mothers' stress levels and indirectly on maternal self-efficacy, in that as maternal stress increased self-efficacy decreased. These findings are similar to a number of other studies aimed at understanding the psychosocial well-being of parents of children with ASD [18–21, 23]. Parenting self-efficacy has also been found to be influenced by other parental characteristics, such as coping style, and guilt [25–28]; future research should attempt to tease apart which parental characteristics are most influencing on both child behavior problems and parenting self-efficacy. 

Additionally, the current study found support for the partial mediating role of parenting self-efficacy between parenting stress and maternal anxiety/depression. The findings indicated that parenting stress was associated with decreased parenting self-efficacy, which in turn accounted for increases in self-reported maternal anxiety/depression. Again, due to its positive association with parenting stress, the child's diagnosis was used as a covariate to diminish any construct overlap and/or artificial effects. Even with the introduction of the child's diagnosis as a covariate, the relationships remained significant.

The current study's support for the partial mediating role of parenting self-efficacy between parenting stress and maternal anxiety/depression adds to Hastings and Brown [29] findings that self-efficacy mediates the relationship between child behavior problems and parental anxiety/depression and provides a richer understanding of how parenting self-efficacy functions in relation to other parental characteristics and behaviors. However, unlike Hastings and Brown [29] findings, the current study only found support for the role of parenting self-efficacy as a partial mediator. In their study of 26 mothers and 26 fathers, Hastings and Brown [29] operationalized parenting self-efficacy to include the parent's perception of their efficacy in relation to their child's problem behaviors. Specifically, parents were asked to rate feelings of competence, their control and satisfaction in dealing with problem behaviors, their perception of how positively they impact their child's problem behaviors, and how difficult they find their child's problem behaviors. However, the PSOC self-efficacy subscale used in the current study provides a more global assessment of parenting self-efficacy. Specifically, parents are asked how easy their child's problems are to solve, how they perceive themselves as a model for other parents, how well they meet the expectations of their child, how familiar they perceive themselves to be with the parenting role, and how strong they perceive their parenting skills to be. Obtaining a more direct measure of self-efficacy in relation to child behavior problems may provide a more domain-specific way in which self-efficacy influences child behavior problems.

### 4.3. Limitations

The current study had several limitations. First, the study did not examine the parenting experience of fathers. Although attempted, only a handful of fathers responded to the survey. To date, few studies have examined the differences in parenting experiences of fathers and mothers. Hastings and Brown [29] examined the role of parenting self-efficacy for mothers and fathers and found evidence that its relationship to parenting stress and parental psychopathology differed in mothers and fathers. Studies examining the parenting experience of fathers have found that fathers report less internalizing symptoms than mothers [8, 12]. These studies suggest that the experience is different for mothers and fathers of children with ASD. Therefore, the need still exists to examine these differences in the parenting experience of mothers and fathers of children with ASD. 

Previous research also suggests possible difficulties in using stress measures, particularly the QRS, within the developmental disability population. According to Glidden [45], the QRS in particular is prone to definitional problems, difficulty replicating results, and failure of the measure to converge with measures of similar constructs. While research support for the psychometric properties of the QRS within the ASD population remains unclear, future research could attempt to circumvent this problem by assessing parental stress using a multimethod approach, with self-report and physiological measurements taken within parent-child lab interactions. The multimethod approach may provide a more accurate understanding of how stress is influencing the parenting experience. 

Additionally, the current study exhibited a characteristic that may represent an artifact of the sample in that mothers seemed to evidence an underreporting of depressive/anxiety symptoms. It is unclear why this trend was evident within this sample. It may be explained by services the families are receiving, methodological issues of social desirability in reporting, or self-selection to the study in that high-risk parents may have been less likely to participate. Despite this limitation, there appeared to be enough variability in reports of anxiety/depression in the current sample to detect significant effects. 

Arguably the biggest limitation is that, due to the study's design, we cannot assert that child behaviors cause increases in parenting stress which then leads to decreases in parenting self-efficacy, or that decreases in self-efficacy then cause increases in maternal anxiety/depression. Further, we cannot assert directionality. It is just as plausible that anxiety/depression decreases parenting self-efficacy, which increases parenting stress. Future research should attempt to tease apart causality with experimental designs and directionality with longitudinal designs. 

Additional difficulties exist because of the study's design. The current study was web based. Therefore, participants' self-selection to the study could explain the rather homogeneous sample. Based on the homogeneity of the sample, results should be interpreted with caution and may not be applicable to all mothering experiences of mothers of children with ASD. Further research with more heterogeneous samples is needed to determine if and how mothering experiences may vary across potentially key characteristics (i.e., race and education). 

The study's design also makes it difficult to confirm the child's ASD diagnosis. While the study did attempt to characterize the level of severity of the child based on symptom items endorsed by the child's mother, the study does not provide another means of substantiating the diagnosis. Nonetheless, the severity measure does provide some insight into ASD-related symptoms endorsed within the study's sample. The study does also provide information related to the provider who diagnosed the child as a means to characterize the quality of the diagnosis. Future research should confirm the ASD diagnosis using a widely accepted measure, such as the ADOS. In addition, it may be helpful to also assess the child's cognitive/intellectual ability, so children with observed cognitive or intellectual deficits may be ruled out or the role of their deficits can be examined.

### 4.4. Conclusions

The current study provides support for the role of parenting stress as a mediator between child behavior problems and parenting self-efficacy, and for the role of parenting self-efficacy as a partial mediator between parenting stress and parental depression/anxiety. These findings suggest that child behavior problems may increase parenting stress, which then interferes with parenting self-efficacy and consequently increases feelings of anxiety/depression in mothers of children with ASD. These findings further knowledge about how ASD symptomatology can affect the parenting experience, particularly in terms of the stress associated with raising a child with ASD and the effect of such stress on mothers' perceptions of parental competence.

The hope is that this study sparks an interest in additional studies that clarify these underlying mechanisms and how they work in concert to affect the parenting experience of raising a child with ASD. At present, although strong theories suggest how these variables interact, research has yet to understand the comprehensive and potentially bidirectional nature in which these variables function. Longitudinal, as well as experimental, designs that assess parent-child interactions in the lab would be ideal ways to address this in future studies. Understanding how these variables function and which variables influence parenting stress and subsequent parental anxiety/depression provides critical information needed in the formation of intervention services for families affected by ASD, implying that services should directly assess and target parenting resources/stress, provide parents with skills to increase efficacy, and add interventions for parental mood/anxiety disorders.

## Figures and Tables

**Table 1:** Participant characteristics.

Parent characteristic	Percentage
*Race*	
Caucasian	94.2
African American	2.2
Latino	1.4
Native American	1.4
Asian/Island pacifiers	.7
*Education	
Some college	29.5
Graduate degree	27.5
College degree	25.4
High school degree	10.9
Some graduate studies	6.5

Child characteristics	Percentage

*Gender *	
Male	79.9
Female	19.9
*Diagnosis *	
Autism	56.9
Pervasive developmental disorder: NOS	22.0
Asperger's disorder	21.2
*Sibling with ASD diagnosis *	
No	83.7
Yes	16.1
*Comorbid disorder present *	
Yes	48.9
No	50.9
*Professional who diagnosed child *	
Developmental pediatrician	37.7
Psychologist	25.4
Neurologist	17.4
Psychiatrist	7.2
Other	3.6
Team of Specialists (e.g., psychologist, developmental pediatrician, neurologist, occupational therapist, and speech therapist)	
Other	
Hospital/children's hospital/autism center (e.g., Kennedy Kreiger, Riley's Children's Hospital, Kluge Hospital, Radford University Center for Autism, and VCU Children's Center)	3.6
School	2.2
Primary care physician	1.4
No response	1.4

**Table 2:** Intercorrelations of primary variables of interest.

	DASS-DA	SDQ	PSOC-SE	QRS	Severity
DASS-DA		.062	−.38**	.50**	.03
SDQ			−.02	.27**	.21*
PSOC-SE				−.17*	−.18*
QRS					.12
Severity					

*Significant at  0.05 level.

**Significant at  0.01 level.

Measure abbreviations:

PSOC-SE = self-efficacy,

QRS = parental stress,

DASS-DA = depression/anxiety.

**Table 3:** Series of regression analyses for parenting stress as mediator child behavior problems and parental self-efficacy.

Regressions	Predictors	*R* ^2^	Adjusted *R* ^2^	Model *p *	*F*	*B*	*β*	*t*
Step 1 (predicting parenting self-efficacy)	Child behavior problems	0.025	0.017	0.035	3.36	−.083	−0.157	−1.83*
Step 2 (predicting parenting stress)	Parent age child diag. Child behavior problems	0.137	0.118	0.0005	6.96	0.227	0.237	2.90**
Step 3 (predicting parenting self-efficacy)	Parent age child diag. Parenting stress	0.082	0.061	0.006	3.86	−0.120	−0.217	−2.48**
Step 4 (predicting parenting self-efficacy)	Parent age child diag. (1) Child behavior problems (2) Parenting stress	0.090	0.062	0.008	3.20	−0.051 −0.106	−.096 −0.191	−1.09 −2.11*

*Significant at 0.05 level.

**Significant at 0.01 level.

Note: Parent age and the child's autism spectrum diagnosis were entered into the equations as control variables for steps using parental stress based on the aforementioned ANOVA and correlation (i.e., Steps 2, 3, and 4).

**Table 4:** Analysis of self-efficacy as a mediator between parental stress and parental depression/anxiety.

Regressions	Predictors	*R* ^2^	Adjusted *R* ^2^	*F*	Model *p *	*B*	*β*	*t*
Step 1 (predicting depression/anxiety)	Parental age child diag. Parenting stress	.223	.204	11.75	.0005	.038	.463	5.56**
Step 2 (predicting parenting self-efficacy)	Parental age child diag. Parenting stress	.082	.061	3.86	.006	−.120	−.217	−2.48**
Step 3 (predicting depression/anxiety)	Parenting self-efficacy	.093	.086	12.79	.0005	−.045	−.306	−3.58**
Step 4 (predicting depression/anxiety)	Parental age child diag.(1) Parenting stress (2) Parenting self-efficacy	.277	.253	11.59	.0005	.033 −.039	.41 −.26	4.93** −3.26**

*Significant at 0.05 level.

**Significant at 0.01 level.

Note: Parent age and the child's autism spectrum diagnosis were entered into the equations as control variables for steps using parental stress based on the aforementioned ANOVA and correlation.
